# Whole-transcriptome analysis of differentially expressed genes in the ray florets and disc florets of *Chrysanthemum morifolium*

**DOI:** 10.1186/s12864-016-2733-z

**Published:** 2016-05-25

**Authors:** Hua Liu, Ming Sun, Dongliang Du, Huitang Pan, Tangren Cheng, Jia Wang, Qixiang Zhang, Yike Gao

**Affiliations:** Beijing Key Laboratory of Ornamental Plants Germplasm Innovation & Molecular Breeding, National Engineering Research Center for Floriculture, Beijing Laboratory of Urban and Rural Ecological Environment and College of Landscape Architecture, Beijing Forestry University, Beijing, 100083 China

**Keywords:** *Chrysanthemum morifolium*, Ray florets, Disc florets, Transcriptome, RNA-seq, Differentially expressed genes, Flower development, Anthocyanin biosynthetic pathway

## Abstract

**Background:**

*Chrysanthemum morifolium* is one of the most important global cut flower and pot plants, and has been cultivated worldwide. However, limited genomic resources are available and the molecular mechanisms involved in the two morphologically distinct floret developmental cycles in chrysanthemum remain unclear.

**Results:**

The transcriptomes of chrysanthemum ray florets, disc florets and leaves were sequenced using Illumina paired-end sequencing technology. In total, 16.9 G reads were assembled into 93,138 unigenes with an average length of 738 bp, of which 44,364 unigenes showed similarity to known proteins in the Swissprot or NCBI non-redundant protein databases. Additionally, 26,320, 22,304 and 13,949 unigenes were assigned to 54 gene ontology (GO) categories, 25 EuKaryotic Orthologous Groups (KOG) categories, and 280 Kyoto Encyclopedia of Genes and Genomes (KEGG) pathways, respectively.

A total of 1863 differentially expressed genes (DEGs) (1210 up-regulated and 653 down-regulated) were identified between ray florets and disc florets, including genes encoding transcription factors and protein kinases. GO and KEGG pathway enrichment analyses were performed on the DEGs to identify differences in the biological processes and pathways between ray florets and disc florets. The important regulatory genes controlling flower development and flower organ determination, as well as important functional genes in the anthocyanin biosynthetic pathway, were identified, of which two leucoanthocyanidin dioxygenase-encoding genes showed specific expression in ray florets. Lastly, reverse transcription quantitative PCR was conducted to validate the DEGs identified in our study.

**Conclusions:**

Comparative transcriptome analysis revealed significant differences in patterns of gene expression and signaling pathways between ray florets and disc florets in *Chrysanthemum morifolium*. This study provided the first step to understanding the molecular mechanism of the differential development of ray florets and disc florets in chrysanthemum, and also provided valuable genomic resources for candidate genes applicable for the breeding of novel varieties in chrysanthemum.

**Electronic supplementary material:**

The online version of this article (doi:10.1186/s12864-016-2733-z) contains supplementary material, which is available to authorized users.

## Background

*Chrysanthemum morifolium* is one of the most important global cut flower and pot plants, and has been cultivated worldwide [1]. Breeders have attempted to breed more chrysanthemum varieties with novel flower types and colors through cross breeding and transgenic biotechnology [1]. Capitulum is the main ornamental part of *C. morifolium*, and the typical inflorescence structure of chrysanthemum is composed of two morphologically distinct florets; namely, ray florets and disc florets. Ray florets are ligulate and zygomorphic, with a showy corolla (petals) and aborted stamens, and play an important role in attracting pollinators. The central disc florets with fertile pollens are radially symmetrical and hermaphroditic, and are used for reproduction in chrysanthemum (Additional file 1). However, the molecular mechanism involved in the development of two morphologically distinct florets has not been well-characterized to date due to the lack of genomic information.

In Arabidopsis, significant progress has been made toward understanding the molecular mechanisms involved in flower development [2]. ABCE models have revealed A-class genes that specify sepal identity, A-class genes together with B-class genes that specify petals, B-class genes together with C-class genes that determine stamens, C-class genes that determines carpel identity, and E-class genes that were thought to pre-establish the floral context so that other organ identity genes could function [3, 4]. Many MADS-Box genes including AP1, AP3, PI, and AG, as well as AP2 genes, were shown to be important regulators involved in flower organ specification [4, 5]. In chrysanthemum, only a few transcription factors in the flower developmental pathway were isolated and analyzed, such as homologs of *APETALA1*, *SEPALLATA3*, and *FRUITFULL* [6].

Flavonoids are ubiquitous secondary metabolites that have important functions in plant physiology [7]. Anthocyanin is a class of flavonoids responsible for the orange-to-blue colors identified in many flowers, leaves, fruits, seeds, and other tissues, and it can attract pollinators and protect against damage from UV irradiation [8]. The production of anthocyanins provide attractive colors for chrysanthemums and improve the ornamental value of chrysanthemum, especially for cut flowers that require bright colors to decorate flower bouquets and baskets. As another class of abundant flavonoids, flavonols also have diversified functions in the plant kingdom, e.g. as effective UV filters, as regulators of auxin transport, as phytoalexins, or as pigments in flowers and fruit [9, 10]. Due to the significant differences in corollaceous colors between ray florets and disc florets of chrysanthemum, it is necessary to perform the comparison of pigments between them. And the molecular mechanism for different flower colors of ray florets and disc florets should be further explored.

In addition, transcriptome profiling of chrysanthemum ray florets was performed using RNA-Seq to investigate light-induced anthocyanin biosynthesis [11]. Interestingly, in chrysanthemum, the ray florets of many varieties exhibit bright colors, such as red, pink, and purple. However, the disc florets show only yellow pollen. The molecular mechanism controlling the flower color difference between ray florets and disc florets remains unknown.

Transcription expression profiling, data assembly, and analysis increase our understanding of the gene regulation networks and biological pathways, can reveal genes downstream of key transcription factors involved in related pathways or networks, and may ultimately explain specific processes [12]. The use of RNA-Seq in studies of chrysanthemum and related species has been demonstrated [11, 13–17]. However, at this time no comparative transcriptome information on the ray florets and disc florets of chrysanthemum has been reported. Here, transcriptional sequencing and comparative analysis of chrysanthemum ray florets, disc florets, and leaves were performed using Illumina assembly technology and RNA-Seq quantification analysis. Based on transcriptional sequencing and analysis, we identified DEGs between ray florets and disc florets to reveal important regulators controlling differential development of ray and disc florets and novel genes involved in regulating stamen development in disc florets. Next, we identified important regulatory genes involved in controlling flower development and flower organ identification, as well as important functional genes in the anthocyanin synthesis pathway to create a list of candidate genes for functional analysis of flowering regulation in chrysanthemum. Our study provides valuable genomic resources and candidate genes for the breeding of novel varieties, and increases our understanding of the developmental molecular mechanisms of both ray florets and disc florets in chrysanthemum.

## Results

### Illumina sequencing and assembly

The cDNA libraries of ray florets, disc florets and leaves were sequenced using the IlluminaHiSeq™ 2000 system. After stringent quality checks and data cleaning, 46,952,994, 51,333,542 and 68,328,428 clean reads were generated for ray florets, disc florets and leaves, respectively. The average proportion of clean reads for the library was 96.2 %.

A total of 124,264 contigs were assembled based on the high-quality reads, with a total size of 68,266,284 bp, an N50 of 648 bp and an average contig length of 549 bp. We then constructed scaffolds between the contigs via the paired-end relationships between the reads. A total of 96,942 scaffolds were obtained, with an N50 of 819 bp and an average length of 721 bp. We filled the intra-scaffold gaps and constructed a non-redundant unigene set from three of the assembled datasets using CAP3 software. Finally, a total of 93,138 high-quality unigene sequences were obtained with an average length of 738 bp (Table 1).

**Table 1:** Summary for the chrysanthemum transcriptome

Statistics	Counts	Total length(bp)	N25 (bp)	N50 (bp)	N75 (bp)	Average length (bp)	Longest (bp)	N%	GC%	Annotation counts	Annotation ratio
Contigs	124,264	68,266,284	1,170	648	414	549	10,284	2.3	37.4		
Primary uniGene	96,942	69,860,137	1,493	819	490	721	11,953	2.3	37.4		
Final unigene	93,138	68,748,959	1,535	853	502	738	11,953	2.41	37.32	44,364	47.63 %

### Gene annotation and functional classification

Of the 93,138 unigenes, 44,364 (47.63 % of the total) were aligned to the Nr protain database (date 2014.03) and Swissprot protein database (date 2014.03) using an E-value threshold of < 1e-5, which meant that 48,774 unigenes (52.37 % of the total) had no Swiss-Prot annotation because of the lack of chrysanthemum genome and EST information (Table 1). The average length of the sequences with significant matches was 927 bp, while that of those without matches was 567 bp. The match efficiency was 89.63 % for unigenes longer than 2000 bp. However, the match efficiency decreased to 31.97 % for those between 200 and 600 bp. Therefore, the sequence lengths affected the annotation.

**Fig. 1:**
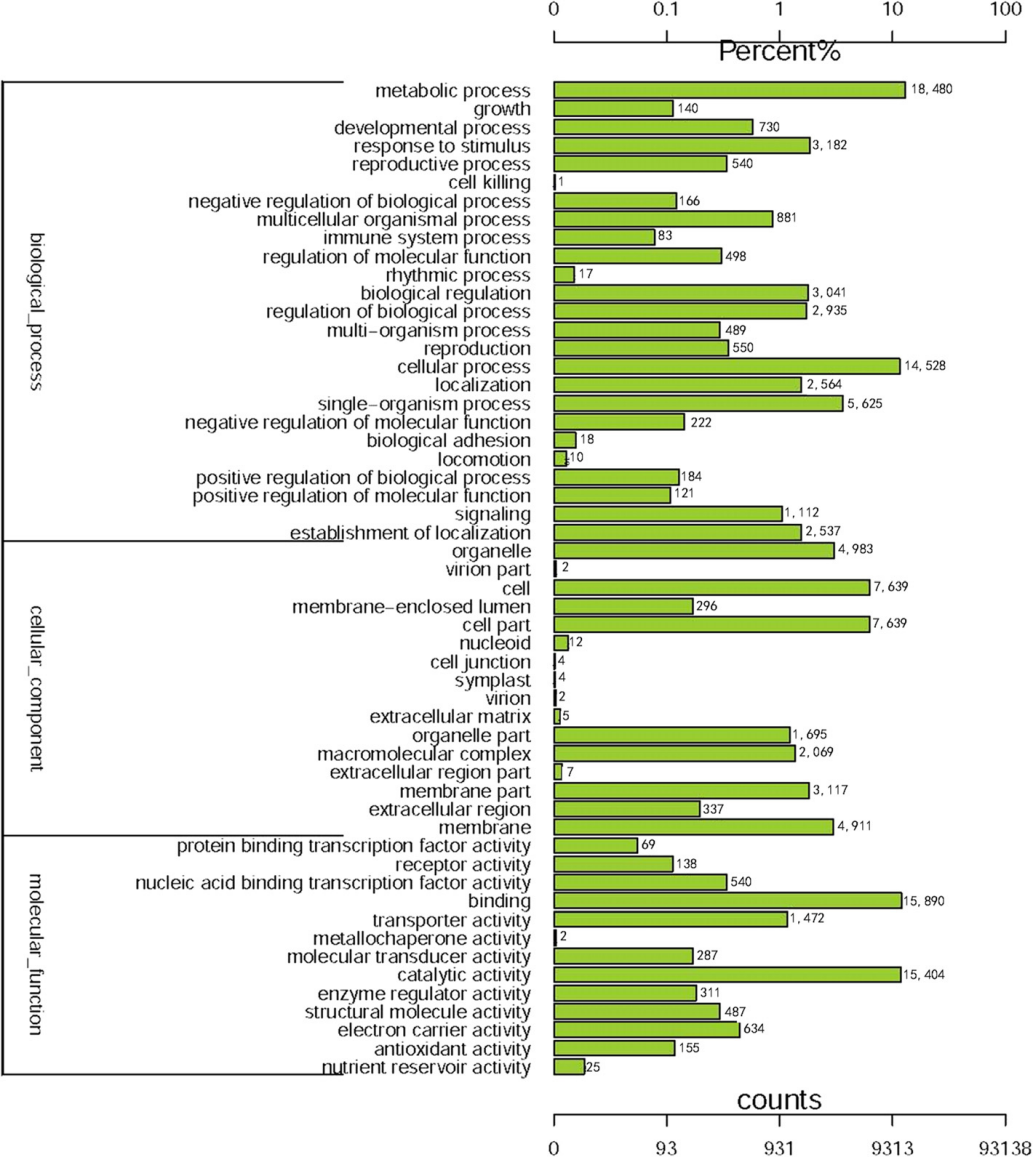
Histogram presentation of Gene Ontology classifications. The results are summarized in three main categories: biological processes, cellular components, and molecular functions. The y-axis on the left side indicates the percentage of genes in a category, and the y-axis on the right side shows the number of genes

Gene Ontology (GO) consisted of three ontologies; molecular functions, cellular components, and biological processes, which facilitated gene annotation and analysis. A total of 26,320 unigenes were classified into 54 functional categories and 19,004 GO terms using the Blast 2GO software, among which 7201 unigenes were assigned to 25 GO categories and 10,282 terms in biochemical processes, 13,285 unigenes were assigned to 13 categories and 5890 terms in molecular functions and 5834 genes were assigned to 16 categories and 2832 terms in cellular component. As shown in Fig. 1, in each of the three main GO classifications, the ‘metabolic process’ (in ‘biological process’), ‘cell part’ (in ‘cell component’), and ‘binding’ (in ‘molecular function’) terms, were dominant, respectively, indicating that numerous metabolic activities were activated during the development of ray and disc florets, which were regulated by a wide range of genes that interacted within cell parts. In addition, we observed a high percentage of genes from the ‘cellular process’, ‘membrane’, and ‘catalytic activity’ categories, but few from ‘cell killing’, ‘cell junction’, and ‘metallochaperone activity’ in each of the three main GO classifications (Fig. 1).

Within the ‘biological process’ category, we identified 25 terms related to flower development in level 6, including ‘flower development’ (GO: 0009908), ‘floral organ development’ (GO: 0048437), ‘regulation of flower development’ (GO: 0009909), ‘floral whorl development’ (GO: 0048438), and ‘photoperiodism, flowering’ (GO: 0048573) (Table 2). In total, 154 unigenes were assigned to these 25 terms, of which 116 were annotated as uncharacterized or predicted proteins and the remainder showed homology to F-box family genes, SET domain protein -coding genes, and GIGANTEA (Additional file 2). In addition, as shown in Table 3, we identified 19 terms that were related to the pollen-pistil interaction, recognition of pollen, pollen development, and stamen development in the ‘biological process’ category. In addition, 295 unigenes were assigned to these 19 terms, of which 166 were annotated as uncharacterized or predicted proteins and the remainder showed homology to serine/threonine-protein kinase and ARK3-like protein coding genes (Additional file 3). These genes assigned to the terms related to flower and stamen development were important candidate genes that may regulate flower development, specification of floral organ identity, and stamen development in chrysanthemum.

**Table 2:** GO terms related to flower development in ‘biological process’ category

Level	GO ID	Term	GO category	No. of the genes assigned to this term
6	GO:0009908	Flower development	Biological process	137
6	GO:0048437	Floral organ development	Biological process	60
6	GO:0009909	Regulation of flower development	Biological process	58
6	GO:0048438	Floral whorl development	Biological process	52
5	GO:0048573	Photoperiodism, flowering	Biological process	34
6	GO:2000028	Regulation of photoperiodism, flowering	Biological process	24
6	GO:0009910	Negative regulation of flower development	Biological process	15
6	GO:0009911	Positive regulation of flower development	Biological process	15
6	GO:0048449	Floral organ formation	Biological process	15
6	GO:0048444	Floral organ morphogenesis	Biological process	15
6	GO:0048586	Regulation of long-day photoperiodism, flowering	Biological process	9
6	GO:0048574	Long-day photoperiodism, flowering	Biological process	9
6	GO:0048579	Negative regulation of long-day photoperiodism, flowering	Biological process	9
6	GO:0010093	Specification of floral organ identity	Biological process	6
6	GO:0010227	Floral organ abscission	Biological process	5
6	GO:0048464	Flower calyx development	Biological process	4
6	GO:0048575	Short-day photoperiodism, flowering	Biological process	3
6	GO:0048587	Regulation of short-day photoperiodism, flowering	Biological process	3
6	GO:0010582	Floral meristem determinacy	Biological process	3
6	GO:0060860	Regulation of floral organ abscission	Biological process	3
6	GO:0060862	Negative regulation of floral organ abscission	Biological process	3
6	GO:0010451	Floral meristem growth	Biological process	2
6	GO:0010080	Regulation of floral meristem growth	Biological process	2
6	GO:0048439	Flower morphogenesis	Biological process	1
6	GO:0048833	Specification of floral organ number	biological process	1

**Table 3:** GO terms related to stamen development in ‘biological process’ category

Level	GO ID	Term	GO category	No. of the genes assigned to this term
6	GO:0009875	Pollen-pistil interaction	Biological process	167
6	GO:0048544	Recognition of pollen	Biological process	165
6	GO:0009555	Pollen development	Biological process	73
6	GO:0048868	Pollen tube development	Biological process	46
6	GO:0009860	Pollen tube growth	Biological process	29
6	GO:0048443	Stamen development	Biological process	25
6	GO:0010208	Pollen wall assembly	Biological process	14
6	GO:0009846	Pollen germination	Biological process	13
6	GO:0010584	Pollen exine formation	Biological process	11
6	GO:0080092	Regulation of pollen tube growth	Biological process	6
6	GO:0010152	Pollen maturation	Biological process	5
6	GO:0048448	Stamen morphogenesis	Biological process	5
6	GO:0048455	Stamen formation	Biological process	4
6	GO:0048235	Pollen sperm cell differentiation	Biological process	3
6	GO:0080110	Sporopollenin biosynthetic process	Biological process	2
6	GO:0010483	Pollen tube reception	Biological process	2
6	GO:0010183	Pollen tube guidance	Biological process	2
6	GO:0009865	Pollen tube adhesion	Biological process	1
6	GO:0060320	Rejection of self pollen	Biological process	1

**Fig. 2:**
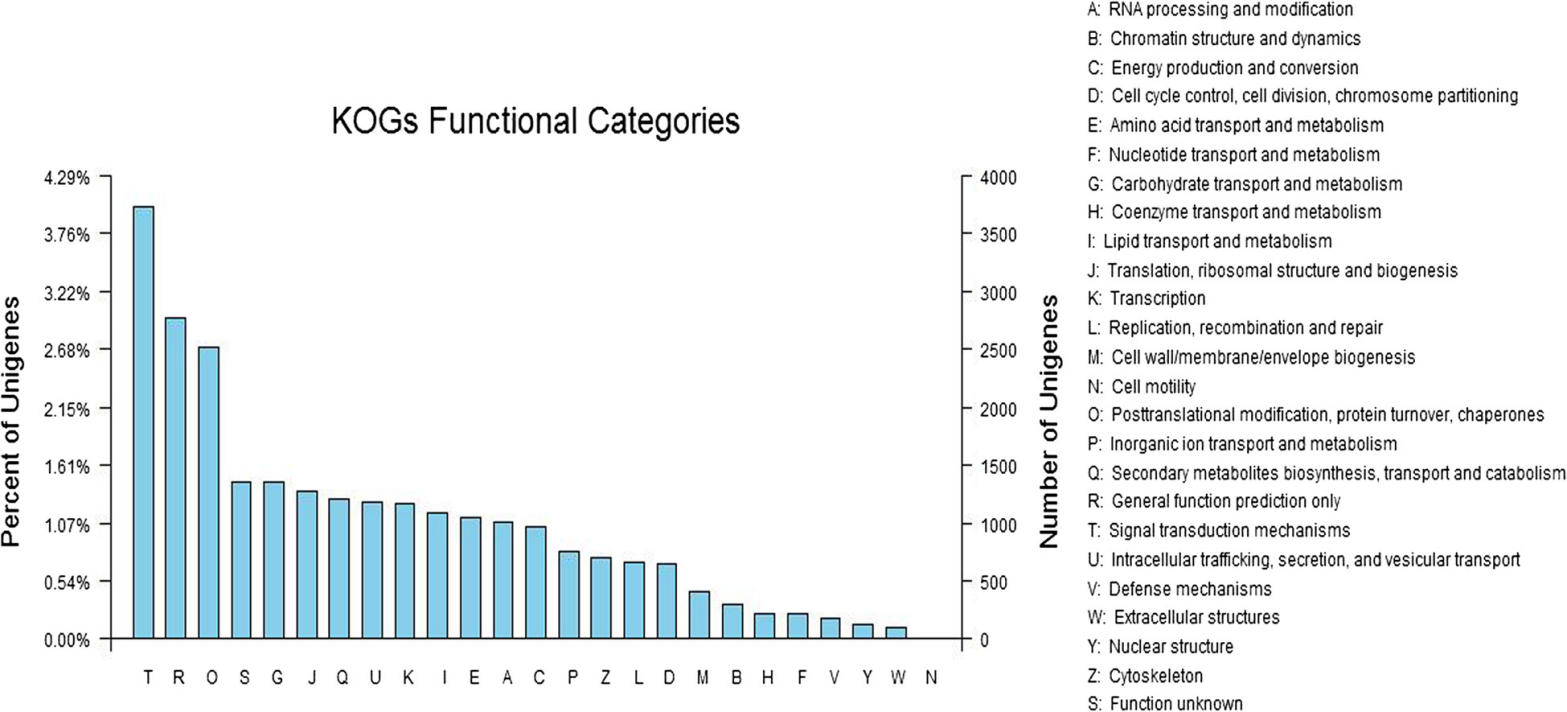
EuKaryotic Orthologous Groups (KOG) classifications in chrysanthemum. A total of 24,321 sequences with KOG classifications within the 25 categories are shown

Eukaryotic Orthologous Groups (KOG) is a eukaryote-specific version of the Clusters of Orthologous Groups (COG) tool, and KOG is typically used to identify orthologous and paralogous proteins, which provide a method of identifying Joint Genome Institute (JGI)-predicted genes based on the KOG classification or ID. To evaluate the completeness of our transcriptome library and the effectiveness of the annotation process, we further searched the annotated sequences for genes involved in KOG classifications. Of 44,364 Nr hits, 22,304 sequences were assigned to the KOG classifications. Among the 25 KOG categories, the cluster for ‘signal transduction mechanisms’ (3739, 16.76 %), ‘general function prediction only’ (2775, 12.44 %), and ‘posttranslational modification, protein turnover, chaperones’ (2519, 11.29 %) represented the largest groups, followed by ‘function unknown’ (1360, 6.10 %), ‘carbohydrate transport and metabolism’ (1354, 6.07 %), ‘translation, ribosomal structure and biogenesis’ (1280, 5.74 %) and ‘secondary metabolites biosynthesis, transport and catabolism’ (1209, 5.42 %). ‘Nuclear structure’ (122, 0.49 %), ‘extracellular structures’ (106, 0.42 %), and ‘cell motility’ (7, 0.028 %) represented the smallest groups (Fig. 2).

The Kyoto Encyclopedia of Genes and Genomes (KEGG) Pathway is a collection of manually drawn pathway maps representing the networks of molecular interactions in the cells and variants of these pathways specific to particular organisms. To further analyze the transcriptome of chrysanthemum, all unigenes were compared with the KEGG database using BLASTx with an E-value threshold of < 1e-5. Of the 44,364 unigenes, 13,949 had significant matches with at least one KEGG pathway in the database and were assigned to 280 KEGG pathways (Table 4). The most represented pathways were ‘metabolic pathways’ (3475 members) and ‘biosynthesis of secondary metabolites’ (1887 members), followed by ‘microbial metabolism in diverse environments’ (741 members), ‘biosynthesis of amino acids’ (482 members), and ‘carbon metabolism’ (471 members). In addition, 381 unigenes were assigned to “plant hormone signal transduction”.

**Table 4:** Categorization of Chrysanthemum unigenes to KEGG biochemical pathways

KEGG Categories	Mapped-ko	Unigene number	Rotio of no.	Parthway-ID
Metabolic pathways	750	3475	24.91 %	ko01100
Biosynthesis of secondary metabolites	346	1887	13.53 %	ko01110
Microbial metabolism in diverse environments	129	741	5.31 %	ko01120
Biosynthesis of amino acids	101	482	3.46 %	ko01230
Carbon metabolism	85	471	3.38 %	ko01200
Starch and sucrose metabolism	34	435	3.12 %	ko00500
Protein processing in endoplasmic reticulum	75	404	2.9 %	ko04141
Plant hormone signal transduction	40	381	2.73 %	ko04075
Plant-pathogen interaction	34	378	2.71 %	ko04626
Cell cycle-yeast	49	372	2.67 %	ko04111
Ribosome	115	371	2.66 %	ko03010
Pyrimidine metabolism	64	366	2.62 %	ko00240
Spliceosome	88	357	2.56 %	ko03040
RNA transport	83	308	2.21 %	ko03013
Epstein-Barr virus infection	59	298	2.14 %	ko05169
RNA degradation	40	256	1.84 %	ko03018
Purine metabolism	76	255	1.83 %	ko00230
Oxidative phosphorylation	79	246	1.76 %	ko00190
Amino sugar and nucleotide sugar metabolism	37	243	1.74 %	ko00520
Ubiquitin mediated proteolysis	54	233	1.67 %	ko04120
Endocytosis	34	226	1.62 %	ko04144
Viral carcinogenesis	41	221	1.58 %	ko05203
mRNA surveillance pathway	47	213	1.53 %	ko03015
Cell cycle	54	206	1.48 %	ko04110
Ribosome biogenesis in eukaryotes	52	170	1.22 %	ko03008
Cysteine and methionine metabolism	34	170	1.22 %	ko00270
HTLV-I infection	39	162	1.16 %	ko05166
Arginine and proline metabolism	38	147	1.05 %	ko00330
Meiosis - yeast	37	139	1 %	ko04113
Non-alcoholic fatty liver disease (NAFLD)	36	105	0.75 %	ko04932
Photosynthesis	36	76	0.54 %	ko00195

### Comparison of the transcriptomes of ray florets and disc florets

#### The set of unigenes common to disc florets and leaves

**Fig. 3:**
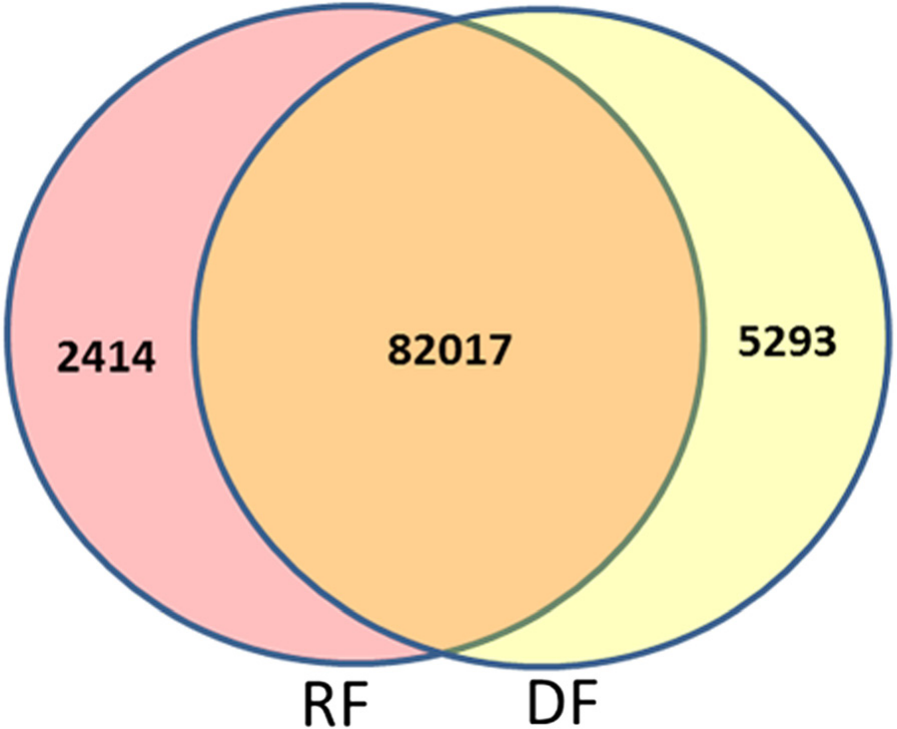
Venn diagram of the number of unigenes with reads per kilobases per million mapped (RPKM) > 0.3 in ray florets (RF), disc florets (DF) and leaves (LE)

The number of unigenes with an RPKM value of > 0.3 that were shared by ray florets and disc florets were 82,017. (Fig. 3). By contrast, 2414 and 5293 unigenes showed specific expression in ray florets and disc florets, respectively. Therefore, more genes were expressed in disc florets than in ray florets, since stamens that were aborted in ray florets developed normally in disc florets.

#### DEGs between ray florets and disc florets

**Fig. 4:**
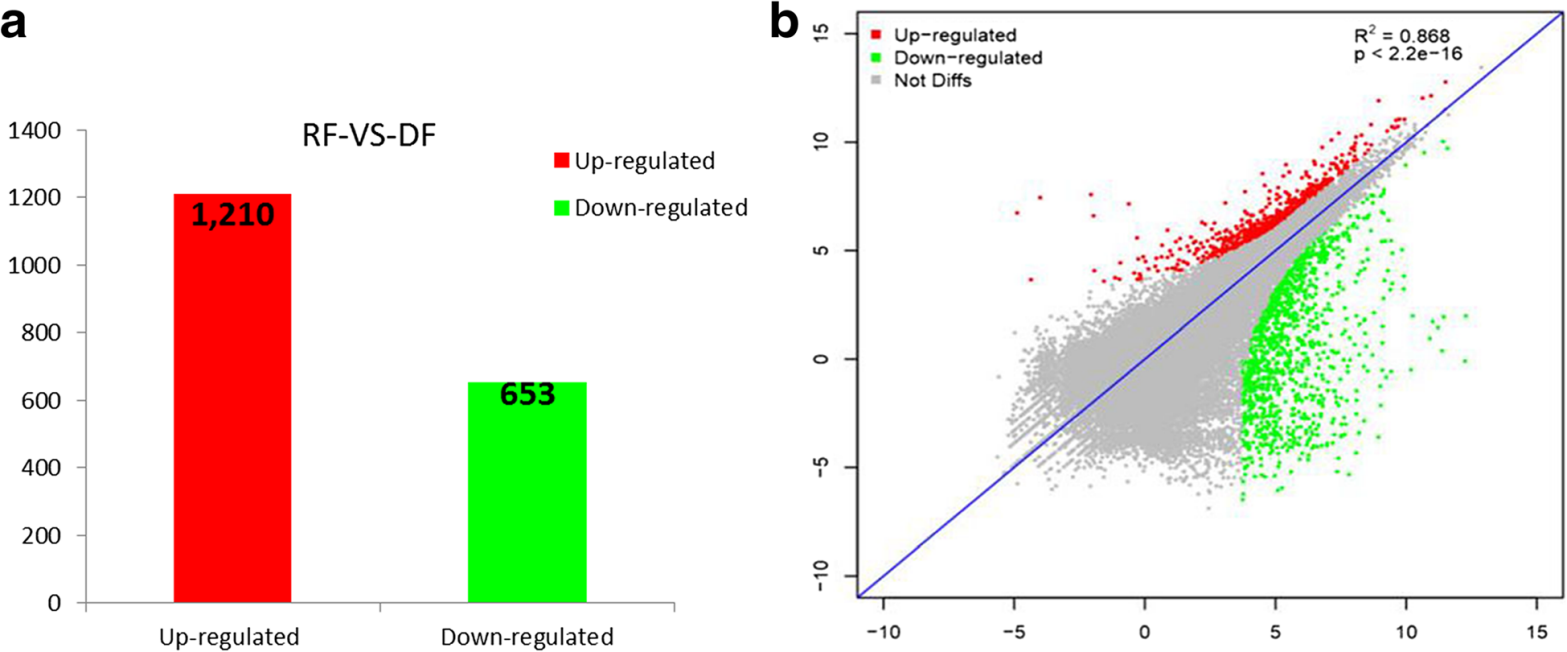
The expression of the gene changes among the different samples. **a** The correlation scatter plot of genes’ expression between ray and disc florets. **b** The number of up-regulated and down-regulated genes between ray florets (RF) and disc florets (DF)

Transcriptomes of ray florets and disc florets were compared, and we mapped the resulting reads to the reference transcriptome. Statistical significance was considered reliable with an RPKM value ≥ 2 in at least one of the three samples. DEGs were filtered with an FDR ≤ 0.05 and a |log2 (ratio)| ≥ 2; only DEGs with a minimum of a two-fold change in expression were used in the analysis of differential gene expression. A total of 1863 DEGs (1210 up-regulated in disc florets and 653 down-regulated in disc florets) were identified between ray and disc florets (Fig. 4a). The correlation of gene expression between ray florets and disc florets was examined using an algorithm developed from the correlation scatter plot (Fig. 4b).

Among the 653 up-regulated unigenes in ray florets relative to disc florets, 247 were annotated to characterized proteins, including many important transcription factors factor-coding genes, functional genes in the anthocyanin biosynthetic pathway, and genes related to pigments or aromatic constituents synthesis (Additional file 4). Of the 1210 genes up-regulated in disc florets relative to ray florets, 387 were annotated to characterized proteins, including some important transcription factors, and several protein kinases (Additional file 5). A total of 132 DEGs were specifically expressed in disc florets relative to ray florets, including WUS and pectinesterase-coding genes, which may be involved in the regulation of stamen development. The annotation information on the DEGs specifically expressed in disc florets was provided in Additional file 6.

GO and KEGG pathway enrichment analyses were performed on the DEGs to identify differences in biological processes and pathways between ray florets and disc florets. In total, 990 DEGs were enriched in 697 GO categories. In the ‘biological process’ category, the dominant terms were the following: ‘oxidation-reduction process’ (GO: 0055114), ‘metabolic process’ (GO: 0008152), and ‘carbohydrate metabolic process’ (GO: 0005975). In the ‘molecular function’ category, the most representative terms were the following: ‘hydrolase activity’ (GO: 0016787), ‘oxidoreductase activity’ (GO: 0016491), and ‘nucleotide binding’ (GO: 0000166). Finally, in the ‘cellular component’ category, the most representative terms were the following: ‘membrane’ (GO: 0016020), ‘integral to membrane’ (GO: 0016021), and ‘cell wall’ (GO: 0005618). Thus, the physiological and biochemical activities involved in oxidation-reduction processes, metabolic processes, and carbohydrate metabolic processes differed between ray florets and disc florets. A total of 399 DEGs were enriched in 204 KEGG pathways. The dominant pathways were the following: ‘metabolic pathways’ (ko01100), ‘biosynthesis of secondary metabolites’ (ko01110), and ‘starch and sucrose metabolism’ (ko00500), which indicated that there were significant differences in physiological and biochemical processes involved in metabolic pathways, biosynthesis of secondary metabolites, as well as starch and sucrose metabolism between ray florets and disc florets. Interestingly, for many of the enriched KEGG pathways, all DEGs were up-regulated in disc florets relative to ray florets, as was the case for ‘phagosome’ (ko04145), ‘arginine and proline metabolism’ (ko00330), ‘progesterone-mediated oocyte maturation’ (ko04914), and many signaling pathways such as ‘oxytocin signaling pathway’ (ko04921), ‘PI3K-Akt signaling pathway’ (ko04151), ‘Rap1 signaling pathway’ (ko04015), ‘neurotrophin signaling pathway’ (ko04722), and ‘Hippo signaling pathway’ (ko04390) (Table 5). Therefore, these signaling pathways may play important roles in disc floret development. The enriched GO terms and KEGG pathways are listed in Additional files 7 and 8.

**Table 5:** KEGG pathways enriching DEGs all up-regulated in disc florets relative to ray florets

Pathway ID	KEGG_name	No. of DEGs in this pathway	No. of down-regulated genes in disc florets	No. of up-regulated genes in disc florets	No. of DEGs in all pathways
ko04145	Phagosome	15	0	15	399
ko00330	Arginine and proline metabolism	14	0	14	399
ko04914	Progesterone-mediated oocyte maturation	14	0	14	399
ko04921	Oxytocin signaling pathway	13	0	13	399
ko04151	PI3K-Akt signaling pathway	13	0	13	399
ko00240	Pyrimidine metabolism	11	0	11	399
ko04015	Rap1 signaling pathway	10	0	10	399
ko05203	Viral carcinogenesis	10	0	10	399
ko04141	Protein processing in endoplasmic reticulum	9	0	9	399
ko04722	Neurotrophin signaling pathway	9	0	9	399
ko04390	Hippo signaling pathway	8	0	8	399
ko03020	RNA polymerase	8	0	8	399
ko04391	Hippo signaling pathway - fly	7	0	7	399
ko05034	Alcoholism	7	0	7	399
ko00250	Alanine, aspartate and glutamate metabolism	7	0	7	399
ko04721	Synaptic vesicle cycle	7	0	7	399
ko04915	Estrogen signaling pathway	7	0	7	399
ko05166	HTLV-I infection	7	0	7	399
ko04110	Cell cycle	7	0	7	399
ko04966	Collecting duct acid secretion	6	0	6	399
ko00910	Nitrogen metabolism	6	0	6	399
ko00960	Tropane, piperidine and pyridine alkaloid biosynthesis	6	0	6	399
ko04810	Regulation of actin cytoskeleton	6	0	6	399
ko04114	Oocyte meiosis	6	0	6	399
ko04621	NOD-like receptor signaling pathway	5	0	5	399
ko05132	Salmonella infection	5	0	5	399
ko05169	Epstein-Barr virus infection	5	0	5	399
ko00970	Aminoacyl-tRNA biosynthesis	5	0	5	399
ko00310	Lysine degradation	5	0	5	399
ko04510	Focal adhesion	5	0	5	399
ko04115	p53 signaling pathway	5	0	5	399
ko04144	Endocytosis	5	0	5	399
ko04666	Fc gamma R-mediated phagocytosis	5	0	5	399

#### Important transcription factors differentially expressed between ray florets and disc florets

A total of 19 important transcription factors family genes were dramatically differentially expressed between ray florets and disc florets. As shown in Table 6, 13 transcription factor family genes were significantly up-regulated in ray florets relative to disc florets, including nine TCP family members (four TCP-like genes and five CYC-like genes) and four other transcription factor family genes (CRT, AP2/ERF, BHLH and DOF). And three CYC-like genes (*CYC2CM2*, *CYC2CM4*, *CYC3CM1*) showed more than 14-fold higher transcription levels in ray florets compared with disc florets, which indicated that these three CYC-like genes may play important roles during the development of ray florets. By contrast, six transcription factor family genes were remarkably up-regulated in the disc florets relative to ray florets, including MADS-box, WUS, NAC and MYB family genes (Table 6). Interestingly, one WUS family gene (*WUSCL*) was specially expressed in the disc florets. These transcription factors may have important functions in the differential development of ray and disc flowers.

**Table 6:** The transcription factors family genes up-regulated in ray florets relative to disc florets

Gene Name	Protein description	Ray florets (RPKM)	Disc florets (RPKM)	Fold change (Ray florets/Disc florets)	*P* value	FDR
*TCP4CM1*	TCP transcription factor	51.32537	22.64641	2.26638	7.32E-04	3.16E-02
*TB1CM1*	TB1-like TCP family transcription factor	58.123	19.53206	2.975775	7.98E-06	5.53E-04
*TB1CM2*	TB1-like TCP family transcription factor	69.52087	22.57164	3.08001	6.65E-07	5.67E-05
*TCP4CM2*	TCP family transcription factor	53.55369	14.03927	3.814564	1.52E-06	1.21E-04
*CYC2CM1*	Cycloidea-like protein	34.34098	6.553657	5.239971	8.31E-06	5.74E-04
*CYC2CM2*	Cycloidea-like protein	12.8801	0.898433	14.33619	9.35E-04	3.94E-02
*CYC2CM3*	Cycloidea-like protein	34.04214	8.603989	3.956553	6.18E-05	3.58E-03
*CYC2CM4*	Cycloidea-like protein	31.7403	1.943154	16.33442	3.29E-08	3.41E-06
*CYC3CM1*	Cycloidea-like protein	24.99901	1.349609	18.52315	2.79E-06	2.12E-04
*CRTCM1*	CRT binding factor	22.50522	2.756458	8.164544	4.62E-05	2.74E-03
*AP2/ERFCM1*	AP2/ERF transcription factor	45.95466	18.56002	2.476002	7.23E-04	3.14E-02
*BHLHCM1*	BHLH transcriptional factor	25.12099	5.310198	4.730707	3.08E-04	1.48E-02
*DOFCM1*	DOF domain class transcription factor	74.34433	28.87703	2.574515	4.7E-06	3.42E-04
*MADSCM1*	MADS-box transcriptional factor	43.54338391	143.5627459	0.303306	1.44E-13	2.53E-11
*SVPCM1*	SVP1	9.370520676	50.98587557	0.183787	9.76E-08	9.67E-06
*NACCM1*	NAC domain transcription factor	15.97280473	81.4721883	0.196052	7.65E-12	1.20E-09
*MYBRCM1*	MYBR domain class transcription factor	25.99922086	56.33325647	0.461525	8.85E-05	3.74E-04
*NACCM2*	NAC domain-containing protein	0.180698746	32.50059629	0.00556	4.11E-09	4.79E-07
*WUSCL*	Transcription factor WUS	0	13.40661064		9.78E-04	4.09E-05

### Identification of important genes controlling flower development and organ determination in Chrysanthemum

**Fig. 5:**
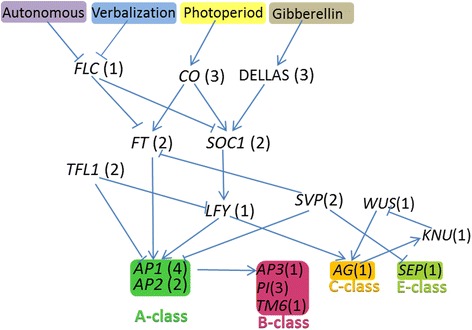
A schematic of flowering regulatory networks in *Chrysanthemum morifolium*. Arrows indicate activation. Bars indicate repression. All homologs of the regulators involved in the photoperiod pathway are listed in Additional file 9. Numbers represent the members of the corresponding genes identified in the chrysanthemum transcriptome

Flowering is a complex process controlled by environmental conditions and developmental regulation. Several signaling pathways involved in flowering control have been identified in Arabidopsis, including the photoperiod pathway, the GA pathway, the vernalization pathway, and the autonomous pathway [18]. These different signaling pathways are known to converge on the regulation of the same floral development genes. As shown in Fig. 5, the gene regulatory networks involved in the different signaling pathways in Arabidopsis were outlined, and the homologs of these important regulators were identified in chrysanthemum (Additional file 9). *FLOWERING LOCUS C* (*FLC*) acts as an inhibitor of flowering, and the genes in the autonomous pathway and vernalization pathway promote flowering by repressing *FLC* expression. One homolog of FLC (*FLCCL*) was identified. FLC represses flowering by repressing the flowering time genes *SOC1* and *FT* (*Flowering Locus T*). *CONSTANS* (*CO*) is a key regulator of the photoperiod response. Both *FT* and *SOC1* (*SUPPRESSOR OF CONSTANS1*) are the early targets of *CO. SOC1* is an upstream regulatory gene of *LEAFY* (*LFY*). *LFY* plays a vital role in the specification of floral meristem identity, and its expression leads to a cascade of transcriptional activities controlling floral meristem development and meristem identity [2]. Based on the protein annotation of transcripts, we identified homologs of *FT* (*FTCL1* and *FTCL2*), *SOC1* (*SOC1CL1* and *SOC1CL2*), *CO* (*COCL1*, *COCL2* and *COCL3*), and *LFY* (*LFYCL*). The A, B, C and E class genes are known to specify flower organ identity. *AP1* and *AP2* are A class genes. However, AP2 is not a MADS-box transcription factor. The transcription of *AP1* is directly activated by *LFY* [19]. Four homologous genes of AP1 (*AP1CL1*, *AP1CL2*, *AP1CL3* and *SQUACL*) and two homologous genes of AP2 (*AP2CL1* and *AP2CL2*) were identified.

In most core eudicot species, B class genes include three different lineages: *PI*, *euAP3* and *TM6*. However, *TM6* like genes seem to have been lost in *Arabidopsis* and *Antirrhinum* [20]. In this study, the homologs of *PI* (*PICL1*, *PICL2*, *GLOCL*), *euAP3* (*AP3CL1*, *AP3CL2*) and *TM6* (*TM6CL*) were identified in *C. morifolium*. We identified one homolog of C-class gene *AG* (*AGCL*) and two homologs of E class gene *SEP* (SEPCL1, SEPCL2) in chrysanthemum. *WUSCHEL* (*WUS*) is known to maintain the stem cell activity. In the floral meristems, WUS binds to adjacent sites of LFY in the AG regulatory region, and both *WUS* and *LFY* promote the up-regulation of *AG* [2]. *KNUCKLES* (*KNU*) encoded a C2H2 zinc finger protein, which is required to repress the expression of *WUS* in the floral meristem, and *WUS* expression disappears by stage 6 in the process of normal floral development [2]. The expression of *KNUCKLES* (*KNU*) is induced by *AG*. One homolog of *WUS* (*WUSCL*) and one homolog of *KNU* (*KNUCL*) were identified. We found that *WUSCL* was specifically expressed in disc florets, which may be important for the differential development of ray and disc flowers. In addition, several other genes known to participate in controlling floral development in chrysanthemum were identified. Annotation information on the unigenes controlling floral development and organ determination in chrysanthemum is provided in Additional file 9.

### Identification of important functional genes in the anthocyanin biosynthetic pathway and pigments in chrysanthemum

**Fig. 6:**
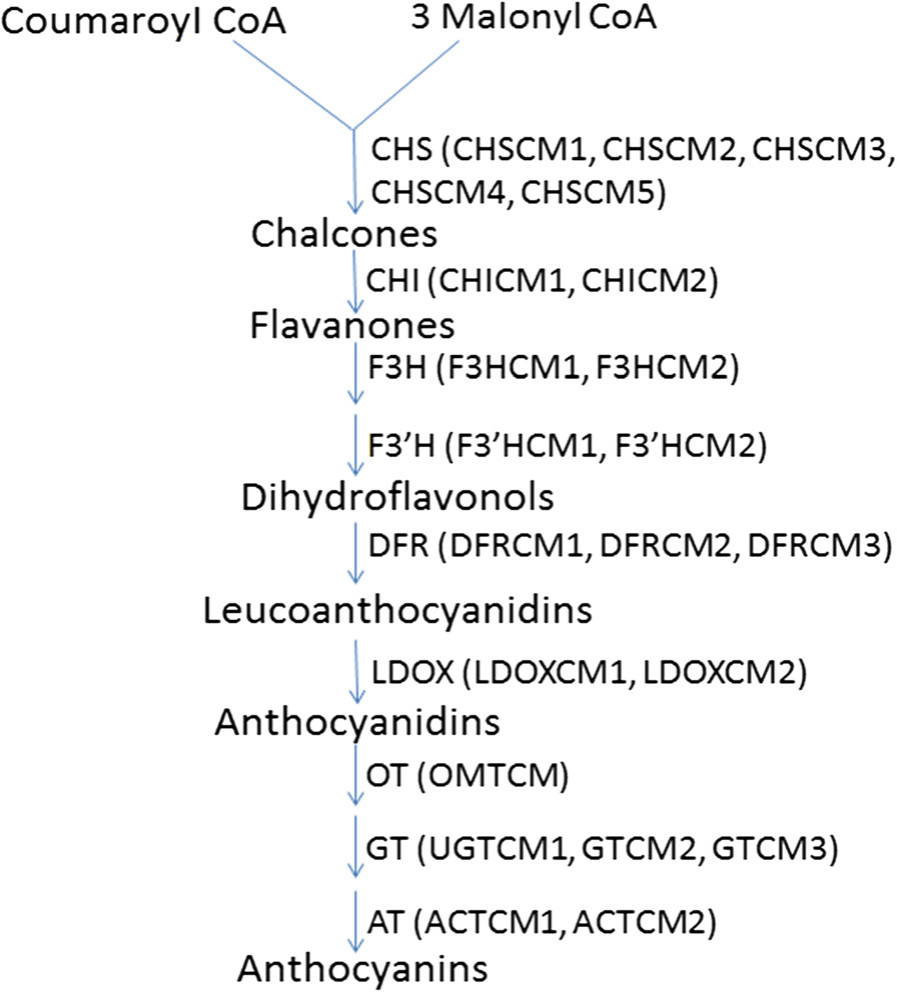
The anthocyanin biosynthetic pathway and the regulatory genes identified in chrysanthemum transcriptome CHS: chalcone synthase, CHI: chalcone isomerase, F3H: flavanone 3-hydroxylase, F3’H: flavonoid 3’-hydroxylase, DFR: dihydroflavonol 4-reductase, LDOX: leucoanthocyanidin dioxygenase, GT: glucosyltransferase, AT: acyltransferase, OT: 3-O-glucoside-6''-O-malonyltransferase

To explore the molecular basis of the difference in flower colors between ray florets and disc florets, we identified important functional genes involved in the chrysanthemum anthocyanin biosynthetic pathway and analyzed the expression differences of these genes between ray florets and disc florets (Fig. 6). As shown in Fig. 6, five chalcone synthase enzyme genes (CHS), two chalcone flavonone isomerase genes (CHI), two flavanone 3-hydroxylase genes (F3H), two flavonoid 3’-hydroxylase genes (F3’H), three dihydroflavonol 4-reductase genes (DFR), two leucoanthocyanidin dioxygenase genes (LDOX), and several glucosyltransferase (GT), 3-O-glucoside-6''-O-malonyltransferase (OT), and acyltransferase (AT) genes were identified in the transcriptome.

The majority of these genes were significantly up-regulated in ray florets relative to disc florets. Only one CHI gene (*CHICM1*), two F3H genes (*F3HCM1* and *F3HCM2*), and two AT genes (*ACTCM1* and *ACTCM2*) were weakly up-regulated in disc florets relative to ray florets. Interestingly two LDOX genes were specifically expressed in the ray florets, which was verified using qRT-PCR (Additional file 10, Fig. 6). Annotation information for these genes in the anthocyanin biosynthetic pathway is listed in Additional file 10.

**Fig. 7:**
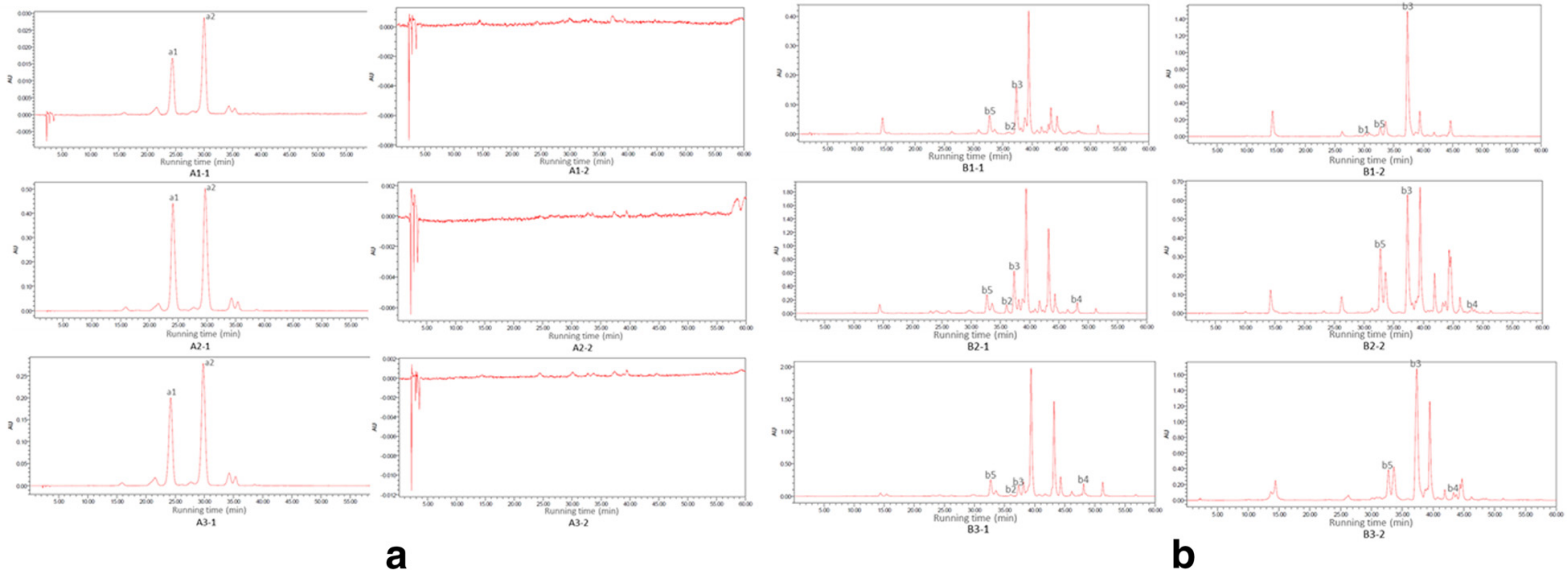
Analysis of pigments in the flowers of pink, red and purple chrysanthemums by HPLC. **a** Analysis of anthocyanin contents in the flowers of pink, red and purple chrysanthemums. **b** Analysis of flavonol contents in the flowers of pink, red and purple chrysanthemums. The x-axis and y-axis indicate the running time and electric signal, respectively. Peak a1 indicates 3-O-6″-O-malonylglucoside (retention time: 24.198). Peak a2 indicates cyanidin 3-O-3″,6″-O-dimalonylglucoside (retention time: 29.988). Peak b1 indicates rutin (retention time: 30.698). Peak b2 indicates kaempferol-3-O-rutinoside (retention time: 35.735). Peak b3 indicates kaempferol-3-O-glucoside (retention time: 37.414). Peak b4 indicates quercetin (retention time: 48.044). Peak b5 indicates quercetin-3-O-glucoside (retention time: 32.699)

The variety of flower colours was determined by the pigment types and contents in flowers. Qualitative analysis of pigments in the flowers of pink, red and purple chrysanthemums was performed by HPLC (Additional file 11). Both anthocyanins and flavonols were detected in the ray florets of pink, red and purple chrysanthemums (Fig. 7). However, no anthocyanins was detected in the disc florets of these flowers above mentioned. (Fig. 7a). As shown in Fig. 7a, the detectable anthocyanins contained cyanidin 3-O-(6 ״-O-malonylglucoside), cyaniding 3-O-(3״,6״ -O-dimalonyl-glucoside) and other unknown anthocyanin contents in the pink, red and purple chrysanthemum ray florets. And the anthocyanin contents in the ray florets of all the three kinds of chrysanthemum were consistent. As shown in Fig. 7b, the flavonols in the ray and disc florets of pink chrysanthemum contained rutin, kaempferol-3-O-rutinoside, kaempferol-3-O-glucoside, quercetin-3-O-glucoside and other unknown flavonol contents; that in the ray and disc florets of red chrysanthemum contained kaempferol-3-O-rutinoside, kaempferol-3-O-glucoside, quercetin, quercetin-3-O-glucoside and other unknown flavonol contents; and that in the ray and disc florets of purple chrysanthemum contained: kaempferol-3-O-rutinoside, quercetin-3-O-glucoside, quercetin, quercetin-3-O-glucoside and other unknown flavonol contents. Therefore, the qualitative analysis of pigments in the flowers of pink, red and purple chrysanthemums indicated no anthocyanins existed in the disc florets of chrysanthemums.

### Verification of the gene expression profiles using qRT-PCR

**Fig. 8:**
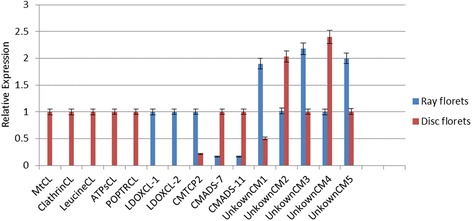
The expression profiles of 15 transcripts in *Chrysanthemum morifolium* by qRT-PCR

To further verify the expression profiles of genes in the Illumina sequencing analyses, 15 unigenes were selected for qRT-PCR using the ray florets and disc florets originally used for RNA-seq. The selected unigenes comprised: two LODX-like genes specifically expressed in the ray florets (*LDOXCL-1* and *LDOXCL*-2), five genes specifically expressed in the disc florets (*MtCL*, *ClathrinCL*, *LeucineCL*, *ATPsCL* and *POPTRCL*), two MADS-Box genes (*CMADS*-7 and *CMADS*-*11*), one TCP gene (*CMTCP2*) and five unigenes coding uncharacterized proteins (*UnkownCM1*, *UnkownCM2*, *UnkownCM3*, *UnkownCM4* and *UnkownCM5*). As shown in Fig. 8, *LDOXCL-1* and *LDOXCL-2* specifically expressed in ray florets; while *MtCL*, *ClathrinCL*, *LeucineCL*, *ATPsCL*, and *POPTRCL* were specifically expressed in disc florets. The RT-PCR results showed that the expression patterns of these 15 genes were consistent with the sequencing data (Additional file 12, Fig. 8).

## Discussion

For the chrysanthemum transcriptome, 93,138 assembly unigenes were obtained, and these unigenes were assigned to a wide range of GO categories, KEGG pathways and KOG classifications, which indicated that a great variety of transcripts are involved in chrysanthemum flower development. We identified unigenes that were annotated to the GO terms related to flower development and stamen development in the ‘biological process’ category, of which many members encoded uncharacterized proteins. These provided important candidate genes for the discovery of novel regulators controlling flower and stamen development in chrysanthemum. Thus, our work provides valuable information and sequence resources for identifying the genes that regulate the development of ray and disc florets in *C. morifolium*.

### Significant differences in gene expression and signaling pathways between ray florets and disc florets form the molecular basis of their morphological and functional distinction

Comparative analysis of the transcriptomes between ray florets and disc florets revealed valuable information of candidate genes and their expression patterns in chrysanthemum. As shown in Fig. 3, ray florets and disc florets shared 82,017 common transcripts, whereas 2414 and 5923 transcripts were specifically expressed in ray florets and disc florets, respectively. More DEGs (1210) were significantly up-regulated in disc florets than (653) in ray florets. Thus, although the majority of unigenes were expressed in ray florets and disc florets, the differences in gene expression at the transcriptome level between ray florets and disc florets were significant. These formed the molecular basis for their morphological and functional distinction.

#### Specific transcription factor genes were predicted to be important regulators during the development of disc florets in chrysanthemum

In this study, we identified several transcription factors genes showing significantly up-regulated expression, including MADS-box, NAC, MYB and WUS transcription factor genes. As master regulators of floral organ determination, MADS-box transcription factors have played important roles during the evolution of flowering plants [5]. Thus, the MADS-box genes up-regulated in disc florets (*MADSCM1* and *SVPCM1*) may have important functions in the development of disc florets. As a major group of plant-specific transcription factors, the NAC family genes play important roles in plant development, including seed development, embryo development, shoot apical meristem formation, fiber development, leaf senescence, and cell division etc. [21, 22]. We identified two NAC family genes (*NACCM1*, *NACCM2*) that showed more than fivefold increased transcript levels in disc florets compared with ray florets, which indicated *NACCM1* and *NACCM2* were important regulators mediating the development of disc florets. MYB transcription factors involved in the ABA-dependent pathway were known to up-regulate abiotic stress-responsive genes [23]. However, one MYB gene (*MYBRCM1*) showed up-regulated expression in disc florets relative to ray florets, indicating that MYB transcription factors probably also play a regulatory role during the development of disc florets. Therefore, besides A, B and C function genes, some other transcription factor (NAC, MYB, WUS) genes may also regulate the development of disc florets in chrysanthemum. Furthermore, these transcription factors genes may have important regulatory roles in the differential development of ray and disc florets, which are required to be explored in the future studies.

#### Many DEGs specifically expressed in disc florets may control pollen development

We identified 132 DEGs that were specifically expressed in disc florets relative to ray florets, among which 30 DEGs were annotated to known proteins, including pectinesterase, copper transporter, metallothionein-like protein, calcium-binding protein Calnexin, profiling, and Amb a 1-like protein, and the others coded proteins of unknown function (Additional file 6). In Arabidopsis, pectinesterase genes are required for pollen separation during normal floral development, which is expressed in anther tissues shortly after meiosis is complete [24]. Thus, it was shown that pectinesterase-coding genes specifically expressed in disc florets may play a vital role in pollen development in chrysanthemum. In Arabidopsis, Copper Transporter COPT1 is expressed in pollen and COPT1 antisense plants showed pollen morphological abnormalities, which suggests that COPT1 is a copper transporter involved in pollen development, and pollen development is one of the most sensitive processes to copper depletion throughout the entire plant [25]. Thus, the classically described symptoms of male sterility caused by pollen defects in plants grown in copper-deficient soils have been accepted [25]. In our study, one copper transporter gene was expressed specifically in disc florets, which indicated that copper transporter genes might be involved in chrysanthemum pollen development. Mmetallothioneins (MTs) consist of a type of low-Mr, Cys-rich proteins, which bind heavy metals and are widely distributed in eukaryotic and prokaryotic organisms [26]. In this study, MTs were predicted to be involved in related signal transduction pathways during the development of stamens in disc florets of chrysanthemums, along with many other DEGs specifically expressed in the disc florets including calcium-binding protein, calnexin, and profiling-coding genes. In addition, three types of pollen allergen, calcium-binding protein, profiling, and Amb a 1-like protein show specific expression in the disc florets, which provides insight into the allergenic potential of chrysanthemum flowers [27].

#### TCP-like genes may mediate the development of ligulate and zygomorphic corolla of ray florets in chrysanthemum

Transcription factors play important roles in the regulation of plant growth and development. As plant-specific transcription factors, TCP transcription factors control cell cycle in angiosperms, resulting in differential growth at meristems and in individual organs; and TCP transcription factors have been proved to regulate morphological traits during plant evolution, including the complex architecture of Asteraceae inflorescence, composed of various types of flowers [28, 29]. Studies in some core eudicots—such as *Antirrhinum majus* (Plantaginaceae), *Helianthus annuus*, *Gerbera hybrida* and *Senecio vulgaris* (Asteraceae) —have shown that CYC2-clade TCPs play prominent roles in establishing flower symmetry by specifying identity to the dorsal (adaxial) region of the flower; and these genes are also involved in the control of flower-type differences in *H. annuus*, *G. hybrida* and *S. vulgaris* of Asteraceae [20, 30–33]. Thus far, little information is available regarding the TCP-like genes in chrysanthemum.

In this study, we identified nine TCP transcription factor family genes that showed higher expression in ray florets relative to disc florets; in particular, the CYC-like genes were significantly up-regulated in ray florets. This result was consistent with studies on CYCLOIDEA/TEOSINTE BRANCHED1 (CYC/TB1) gene family in *G. hybrida* and *H. annuus*, which indicated that all sunflower and gerbera CYC2 clade genes showed up-regulated expression in marginal ray (and trans) flowers. In Asteraceae, previous studies concerning flower symmetry were restricted to CYC2-clade TCPs. However, we also identified other TCP-like genes with up-regulated expression in ray florets of chrysanthemums. Therefore, 1) the up-regulated expression of CYC-like genes contributes to the development of ligulate and zygomorphic corolla in ray florets of chrysanthemum; 2) in addition to CYC2-clade genes, other TCP-like genes may also play a role in the control of flower-type differences in chrysanthemum. Further studies are required to explore the exact roles of these TCP-like genes in the regulation of ray floret development in chrysanthemum.

### A new version of the ABC model for chrysanthemum is required

In the present study, we outlined the gene regulatory networks controlling flowering involved in the different signaling pathways in Arabidopsis and identified the homologs of these important regulators involved in floral meristem identity and the regulation of flower development, including AP2 and many MADS box genes (Fig. 5). The classic ABC model was developed based on floral mutant phenotypes in *Arabidopsis* and *Antirrhinum*, which postulates that three different gene classes (or functions) specify the identity of the flower organs in the four floral whorls and later E-function genes required for all other functions were added to the model [5]. The homologs of A-, B-, C-, and E-class genes were identified based on the protein annotation of unigenes in chrysanthemum, including one homolog of the TM6 lineage (B-class genes), which is not found in *Arabidopsis* and *Antirrhinum*. However, different from the traditional model plants, the capitulum of chrysanthemum is composed of two morphologically distinct florets (ray florets and disc florets). Thus, further studies are required to explore the regulatory mechanism of A, B, and C function genes in chrysanthemum. Studies in gerbera have shown that 1) flower-type specific MADS-box protein complexes play a central role in differential development of ray and disc flowers; 2) B class MADS-box genes; *GGLO1* (*PI/GLO*-like gene) and *GDEF2* (eu*AP3*-like gene), mediate the classical B-function since they determine petal and stamen identities; 3) *GDEF1* (B-class gene of *TM6* lineages) is not involved in determining petal identity, and it may independently regulate stamen development of late stages [20, 34]. In chrysanthemum, *CDM111* (A function gene) is the functional equivalent to *AP1*, and *CDM44* (E function gene) is the functional equivalent to *SEP3* [6]. However, further studies are required to explore the functions of A-, B-, C-, and E-class genes in flower organ determination and differential development of ray and disc florets in chrysanthemum. Furthermore, we identified a further two regulators of flower development together with MADS-box genes; *WUS* (*WUSCL*) and *KNU* (*KNUCL*), whose function in regulation of flower development in Asteraceae remains unclear. WUS, which binds to the adjacent sites of LFY in the AG regulatory region, promotes the up-regulation of AG [2]. KNU functions as an inhibitor of WUS in the floral meristem. Interestingly, we found that WUSCL was specifically expressed in the disc florets. Recently, it has been illuminated that the prototypic WOX-family member, the WUS gene, is a bifunctional transcription factor acting as a repressor in stem-cell regulation and as an activator in floral patterning [35]. The WUS gene is sufficient to return differentiating cells to stem cells to maintain stem cells, and its expression is repressed at stage 6 in an AG-dependent manner [36]. Thus, in our study it was predicted that 1) the specific expression of WUS in the disc floret region contributes to formation of the indefinite inflorescence in chrysanthemum; 2) WUS is involved in flower organ determination together with AG. Further studies are required to explore the functions of WUS in flower development and organ determination in chrysanthemum.

### Anthocyanidins are not synthesized in disc florets as two LDOX genes are not expressed

We analyzed important genes in the chrysanthemum anthocyanin biosynthetic pathway and identified two LDOX genes that are highly expressed in ray florets but are not expressed in disc florets. Leucoanthocyanidindioxygenase (LDOX) is also known as anthocyanidinsynthase (ANS), belongs to the OGD family, and catalyzes the synthesis of corresponding colored anthocyanidins. High expression of LDOX genes in the ray florets promoted anthocyanidin synthesis and accumulation. The qualitative analysis of pigments in the flower of chrysanthemum confirmed that anthocyanins are present only in the ray florets, while the disc florets contained no anthocyanidins, which was consistent with LODX-like gene specific expression in ray florets. Anthocyanins in the ray florets provide vibrant colors as visible signals to attract insects for pollination.

## Conclusions

In our study, comparative transcriptome analysis revealed significant differences in gene expression and signaling pathways between ray florets and disc florets in *C. morifolium*. We identified unigenes that were annotated to the GO terms related to flower development and stamen development in the ‘biological process’ category, which provided important candidate genes for the discovery of novel regulators controlling flower and stamen development in chrysanthemum. We identified homologs of the important regulators of flower development and organ determination, including the homologs of A-, B-, C-, and E-class genes as well as two regulators controlling flower development together with MADS-box genes: WUS (*WUSCL*) and KNU (*KNUCL*). In addition, WUSCL, which is specifically expressed in the disc florets, may play important roles in flower development and organ determination. The important functional genes in the anthocyanin synthesis pathway were analyzed, and we identified two LDOX genes that showed specific expression in ray florets, which explains why anthocyanidins were not synthesized in disc florets. These findings indicate that the differential development of two morphologically distinct florets (ray florets and disc florets) is a complex biological process regulated by a wide range of factors, and further studies are required to explore the gene regulatory networks involved in this process. This study represents the first step in exploring the molecular mechanism of the differential development of ray florets and disc florets in chrysanthemum, and it provided valuable genomic information and candidate genes for the breeding of novel chrysanthemum varieties.

## Methods

### Plant materials and RNA extraction

The tissues (ray florets, disc florets and leaves) used in the study were obtained from a ground-cover chrysanthemum variety (*C. morifolium* ‘Fenditan’, a hybrid of chrysanthemum varieties) cultivated in a greenhouse under an 8-h light/16-h dark cycle at 23 °C in Beijing Forestry University (116.3°E, 40.0°N). After marginal ray florets (central disc florets) of the flower buds were fully formed, about 60–120 marginal ray florets (central disc florets) and 3–4 fully expanded leaves were collected between 9:00–12:00 a.m. once per week until the flowers were in full bloom. The collected plant materials were placed immediately in liquid N2 and stored at −80 °C until RNA extraction. Total RNA was extracted using the RNeasy Plant Mini Kit (Qiagen, Cat. #74904). RNA quantity and quality were assessed using a NanoDrop ND2000 instrument (Thermo Scientific).

### Illumina sequencing, and de novo assembly functional annotation

Ray florets and disc floret development are continuous developmental processes. Therefore, to obtain complete transcriptome information, we pooled equivalent quantities of total RNA isolated from different developmental stages of ray florets and disc florets, and the pooled total RNA samples were used for sequencing. Thus, leaves were collected at the same time as the samples of ray florets and disc florets. In total, 10 μg of total RNA were collected for each sample for sequencing. We conducted llumina sequencing using an Illumina HiSeq™ 2000 system (Illumina, San Diego, CA, USA) in Shanghai (ShoBiotechnology Corporation (SBC), Shanghai, China). After poly (A) mRNA was purified and fragmented into small pieces, the first strand cDNAs were synthesized using random hexamers primers, after which the second strand was synthesized. Double-strand cDNAs were purified with QiaQuick PCR extraction kit (Qiagen) and resolved using EB buffer for end reparation and addition of a poly (A) tail. The short fragments were then connected with sequencing adapters. Briefly, a cDNA library was constructed with average insert sizes of 300–500 bp. We conducted cDNA sequencing using the Illumina HiSeq™ 2000 system according to the manufacturer’s protocols, with paired end 2 × 100 nt multiplex. After removing the low-quality reads, the transcriptome sequences were assembled into distinct contigs using the short reads with the software CLC Genomic Workbench 5.5 software (CLC Bio, Denmark). Scaffolds were then constructed between contigs employing the paired-end relationships between the reads. The intra-scaffold gaps were filled and a non-redundant unigene set was constructed from all three assembled datasets using CAP3 software [37].

For functional annotation, BLASTX alignment (E-value of 1.00E-5) between unigenes and protein databases, including Nr (non-redundant protein database, NCBI), Swiss-Port (http://www.expasy.ch/sprot), KEGG (http://www.genome.jp/kegg), and KOG (http://www.ncbi.nlm.nih.gov/KOG) was performed, and the sequence direction of unigenes was determined based on the optimal alignment results. GO annotations for the unigenes were obtained using the Blast2GO software [38].

### Analysis of chrysanthemum transcriptome sequencing results

The expression level of each unigene was calculated using RNA-Seq quantification analysis as the number of reads per kilobase of exon model per Million mapped reads (RPKM) [39]. The chrysanthemum transcriptome from the three samples was employed as reference for the screening and analysis of differentially expressed unigenes due to the unavailability of existing data. A rigorous algorithm was used to identify differentially expressed genes based on the method of Audic et al. [40]. We used the false discovery rate (FDR) to confirm the threshold of the P-value in multiple tests and analyses [41]. An FDR of < 0.05 and the absolute value of log2 (ratio) ≥ 2 were used as thresholds to define significant differences in gene expression [39]. Only the differentially expressed genes (DEGs) with a minimum of a twofold change in expression were used in the analysis of gene differential expression.

### Gene expression analysis based on qRT-PCR

Total RNA was extracted from the ray florets, disc florets, and leaves as described above. Total RNA was treated with DNase (Promega, USA), and then subjected to reverse transcription to cDNA using a reverse transcription system (Tiangen, China). Real-time RT-PCR was performed using the PikoReal real-time PCR system (Thermo Fisher Scientific, Germany). Each reaction was performed in a total volume of 20 μL which contained 2 μL first-strand cDNA as template, with an amplification program of 30 s at 95 °C, followed by 40 cycles of 5 s at 95 °C and 30 s at 60 °C. Gene-specific primers shown in Additional file 11, were used to perform the relative quantification of each gene. All real-time RT-PCR experiments were conducted in three biological replicates. Each replicate was measured in triplicate. The relative expression levels were analyzed using the 2-ΔΔCt method, with the protein phosphatase 2A (*PP2Acs*) gene of *C. morrifolium* as the reference gene [42].

### Qualitative analysis of pigments in chrysanthemum flower

The analysis of anthcyanin profiles was carried out using high pressure liquid chromatography (HPLC). 0.15-0.18 g sample was ground into fine powder in liquid N2, and then homogenized in 1.8 ml anthocyanin extracts [methanol: distilled water: methane acid: trifluoroacetic acid (70:27:2:1, v/v/v/v)] assisted by sonication at 20 °C for 30 min [43]. Then, the mixture was centrifuged at 16000 × g for 10 min at 20 °C, and the supernate was passed through a 0.22 μm reinforced nylon membrane filter (Shanghai ANPEL, Shanghai City, China) before injection. The HPLC system was consisted of a Waters 2695 separation module with a 996 photodiode array detector (PDA) controlled by an Empower 2 workstation (Waters, Milford, MA). A X Bridge BEH C18 column (150 × 4.6 mm, 2.5 μm, Waters, Milford, MA) was used to separate the anthocyanins and flavonols. The column was maintained at 25 °C and water containing 0.5 % (v/v) formic acid (A) and acetonitrile (B) were used as the mobile phase. Gradient elution was applied at a flow rate of 0.5 mL/min with the following conditions: 0 min, 95 %A + 5 %B, 5 min, 90 %A + 10 %B, 30 min, 81 %A + 19 %B, 50 min, 60 %A + 40 %B. 50.01-60 min, 95 %A + 5 %B. The injection volume was 10 μL and the photodiode array detector (PDA) was set at 520 nm for anthocyanin and 350 nm for flavonols [44]. Three biological replicates were analyzed for each sample type.

## Additional files

Additional file 1: Flowers of chrysanthemum A The capitulum. B The ray floret and disc floret. (TIF 2355 kb) Additional file 2: The genes assigned to GO terms related to flower development in chrysanthemum. (XLSX 18 kb) Additional file 3: The genes assigned to GO terms related to stamen development in chrysanthemum. (XLSX 28 kb) Additional file 4: The up-regulated genes in ray florets in chrysanthemum. (XLSX 94 kb) Additional file 5: The down-regulated genes in ray florets in chrysanthemum. (XLSX 153 kb) Additional file 6: The DEGs specifically expressed in disc florets. (XLSX 23 kb) Additional file 7: GO enrichment of DEGs between ray florets and disc florets. (XLSX 73 kb) Additional file 8: KEGG enrichment of DEGs between ray florets and disc florets. (XLSX 27 kb) Additional file 9: The important genes controlling flower development and organ determination in chrysanthemum. (XLSX 12 kb) Additional file 10: The important function genes in anthocyanin biosynthetic pathway in chrysanthemum. (XLSX 11 kb) Additional file 11: Flowers of chrysanthemum used in analysis of pigments by HPLC. A The ray and disc florets of pink chrysanthemum. B The ray and disc florets of red chrysanthemum. C The ray and disc florets of purple chrysanthemum. (TIF 609 kb) Additional file 12: Primers used in real-time quantitative PCR of *Chrysanthemum morifolium. (XLSX 10 kb)*
