# Supplementary figures and images for: Whole-transcriptome analysis of differentially expressed genes in the ray florets and disc florets of Chrysanthemum morifolium
Source: BMC Genomics. 2016 May 25;17:398. doi: 10.1186/s12864-016-2733-z (PMC4881213; doi:10.1186/s12864-016-2733-z)

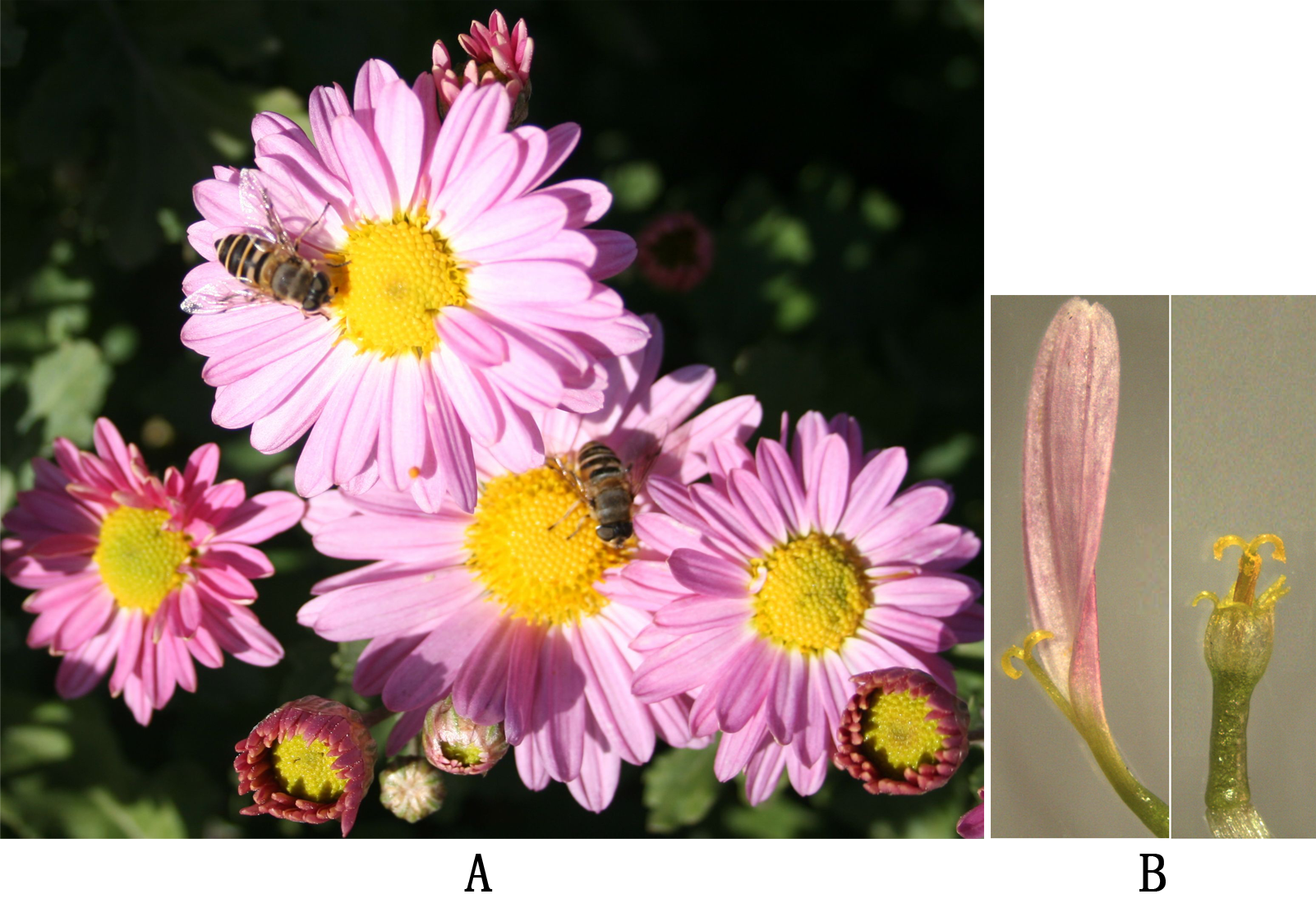

Supplement: Additional file 1: — Flowers of chrysanthemum A The capitulum. B The ray floret and disc floret. (TIF 2355 kb) [file 12864_2016_2733_MOESM1_ESM.tif]

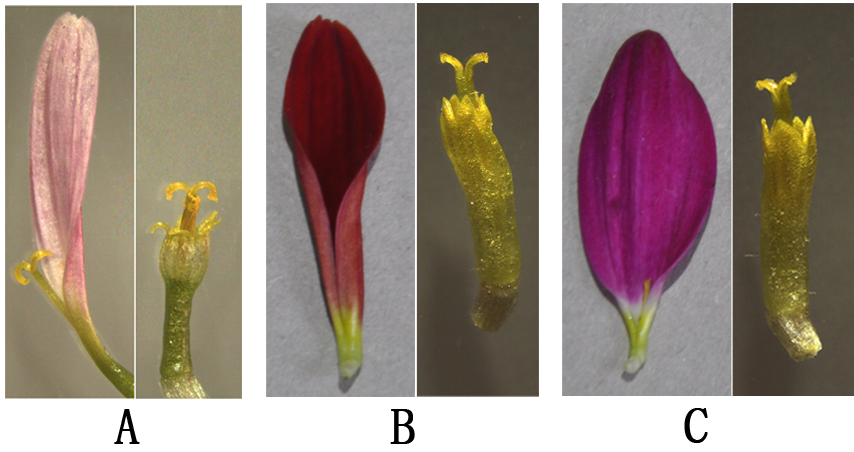

Supplement: Additional file 11: — Flowers of chrysanthemum used in analysis of pigments by HPLC. A The ray and disc florets of pink chrysanthemum. B The ray and disc florets of red chrysanthemum. C The ray and disc florets of purple chrysanthemum. (TIF 609 kb) [file 12864_2016_2733_MOESM11_ESM.tif]
